# Radiation‐Sensitive Dendrimer‐Based Drug Delivery System

**DOI:** 10.1002/advs.201700339

**Published:** 2017-12-05

**Authors:** Szu‐Yuan Wu, Hsiao‐Ying Chou, Chiou‐Hwa Yuh, Shewaye Lakew Mekuria, Yu‐Chih Kao, Hsieh‐Chih Tsai

**Affiliations:** ^1^ Department of Radiation Oncology Wan Fang Hospital Taipei Medical University 116 Taipei Taiwan; ^2^ Department of Internal Medicine School of Medicine College of Medicine Taipei Medical University 110 Taipei Taiwan; ^3^ Graduate Institute of Applied Science and Technology National Taiwan University of Science and Technology 106 Taipei Taiwan; ^4^ Institute of Molecular and Genomic Medicine National Health Research Institutes 350 Zhunan Miaoli Taiwan; ^5^ Institute of Bioinformatics and Structural Biology National Tsing Hua University 300 Hsinchu Taiwan; ^6^ Department of Biological Science and Technology National Chiao Tung University 300 Hsinchu Taiwan

**Keywords:** combination therapies, dendrimers, doxorubicin, HeLa cells, zebrafish

## Abstract

Combination of chemotherapy and radiotherapy is used to enhance local drug delivery while reducing off‐target tissue effects. Anticancer drug doxorubicin (DOX) is loaded into l‐cysteine modified G4.5 dendrimer (GC/DOX) and released at different pH values in the presence and absence of γ‐radiation. Presence of γ‐radiation significantly improves DOX release from the GC/DOX under acidic pH conditions, suggesting that GC dendrimer is a radiation‐sensitive drug delivery system. GC/DOX is further evaluated by determining cytotoxicity in uterine cervical carcinoma HeLa cells. GC/DOX shows high affinity for cancer cells and effective drug release following an external stimulus (radiation exposure), whereas an in vivo zebrafish study confirms that l‐cysteine acts as a radiosensitizer. GC/DOX treatment combined with radiotherapy synergistically and successfully inhibits cancer cell growth.

## 1 Introduction

Radiotherapy is widely used for cancer treatment, as γ‐radiation alters molecular structure and biological properties of biomolecules.^1^ Radiation affects cells directly by damaging DNA and indirectly by exciting water molecules and producing free radicals. Exposure to γ‐radiation mediates the lysis of water, generating reactive oxygen species (ROS) and causing functional deficiency and apoptosis of cells.^2^, ^3^, ^4^ In certain tumors, cancer cell survival rates after radiotherapy are high (e.g., early‐stage laryngeal cancer and nonsmall cell lung cancer).^5^ Patients with these tumors should be treated with combined radiation and chemotherapy because of synergistic interaction of radiotherapy and chemotherapeutic drugs.^6^ Combined use of radiotherapy and anticancer drugs including doxorubicin (DOX) was shown to additively inhibit proliferation of cancer cells.^7^ DOX treatment combined with γ‐radiation resulted in a 1.4‐fold increase in tumor cell death compared to γ‐radiation alone.^8^ Liposomal DOX (Caelyx) administration with chemoradiation induced synergistic effects such as improved intratumoral drug uptake and distribution, resulting in enhanced antitumor effects.^9^ Additionally, DOX treatment combined with radiation therapy was shown to generate a greater amount of ROS and inhibit tumor growth more effectively than other treatment methods studied.^10^, ^11^, ^12^ However, systemic chemotherapy remains toxic to healthy tissues and is characterized by low drug concentration at tumor sites.^13^ To address these issues, polymer carriers have recently been used to regulate the amount of drugs released within cancer cells.^14^, ^15^, ^16^, ^17^ Among these carriers are dendrimers, extensively branched polymers that provide a unique platform for constructing various biocompatible multifunctional systems for drug delivery applications.^18^, ^19^, ^20^

As radiation therapy includes ionizing radiation, it may damage suspected sites of residual disease.^21^ Sulfhydryl bond in the structure of l‐cysteine (l‐Cys) allows it to act as a radio protector by scavenging free radicals.^22^, ^23^, ^24^ Experiments in rats demonstrated that animals administered l‐Cys withstood normally lethal doses of X‐rays^25^ and showed considerably reduced damage to essential organs compared to untreated animals.^26^ In addition, cysteine‐containing polypeptides form disulfide cross‐links, which are useful in drug carrier design,^27^ as reduction of disulfide bonds can be used to trigger the release of anticancer drugs.^28^ Disulfide cross‐linking has been combined with internal and external stimuli for drug delivery. Stimuli‐responsive biomaterials have been developed and are increasingly used for controlled drug delivery.^29^ The G4.5 polyamidoamine (PAMAM) dendrimer is a highly branched, biocompatible, and monodispersive fourth‐generation polymer with a variable pH‐dependent conformation, symmetric branches, and 128 surface carboxylate groups, and has been widely used in drug delivery systems.^30^ Size and charge of PAMAM dendrimers affect cytotoxicity and biodistribution,^31^ with anionic dendrimers displaying significantly less cytotoxicity.^32^

In this study, in order to provide localized chemo‐ and radiation therapy, we designed a drug carrier responsive to external stimuli. High‐energy ionizing γ‐radiation penetrates deep in suspect tumor tissue targeting cancer cells.^4^ Tissue penetration of γ‐radiation allows it to be used as an external stimulus, triggering localized release of drugs.^33^ In this study, we aimed to develop a radiation‐sensitive drug‐delivery system based on activation of G4.5‐Cys (GC) after exposure to a low dose of γ‐radiation (5 Gy), which would trigger the cleavage of disulfide bonds. Decorating the dendrimer with radiosensitizer l‐Cys would improve targeting of tumor tissues. Biocompatible G4.5 PAMAM dendrimer was selected as the basis of the system as it was previously conjugated with l‐Cys to evaluate drug release under γ‐radiation.^34^ However, few studies have focused on the use of dendrimer carriers in cancer radiation therapy and to our knowledge this study is the first to use l‐Cys as a radiosensitizer in such drug‐delivery systems.

## 2 Results and Discussion

### 2.1 Structural Analysis of Multifunctional Dendrimer Derivative

**Scheme 1:**
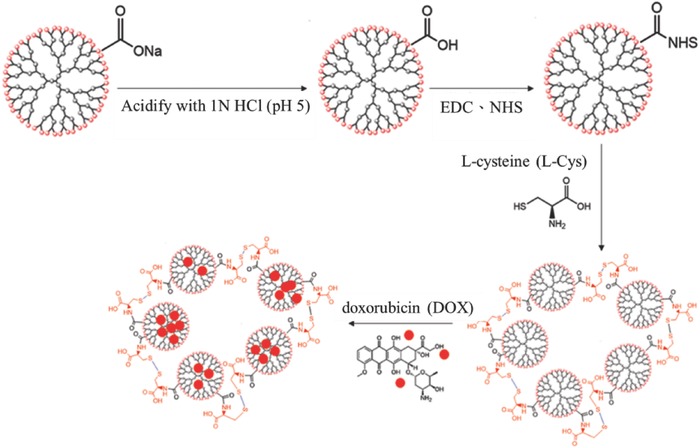
Synthesis and DOX loading of the GC nanoparticle.

First step in evaluating the drug release kinetics of dendrimer derivatives in cancer cells in the presence of γ‐radiation was conjugation of the G4.5 dendrimer with l‐Cys. GC particles were synthesized by forming amide bonds between activated G4.5 carboxylic groups and amino groups of l‐Cys, in the presence of 1‐(3‐(dimethylamino)propyl)‐3‐ethylcarbodiimide hydrochloride (EDC) and N‐hydroxylsuccinimide (NHS), allowing free thiol groups to form disulfide bonds and dendrimer nanoparticles, as shown in **Scheme** **1**. Synthesized GC nanoparticles were loaded with DOX to obtain anticancer drug delivery systems.

**Figure 1:**
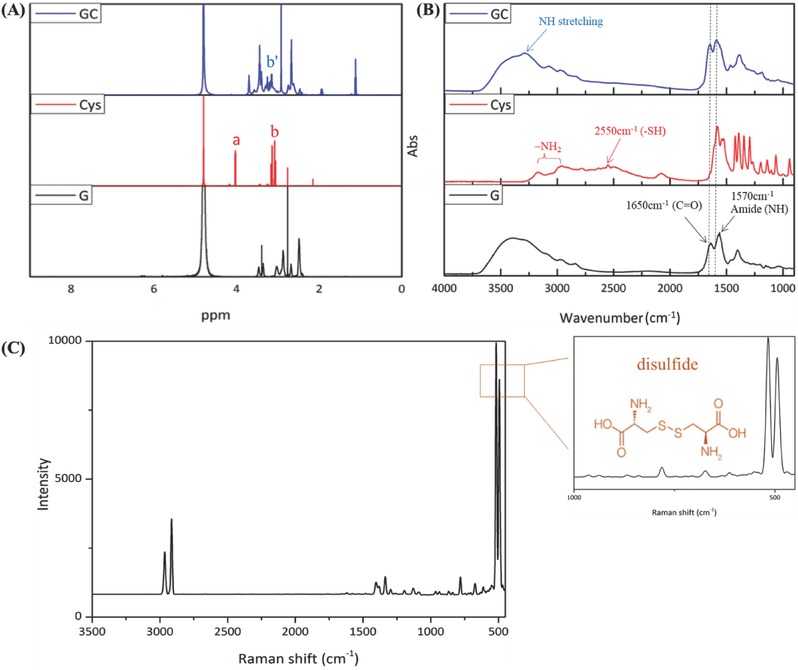
G4.5, l‐Cys, and GC spectra. A) ^1^H NMR spectra, B) FT‐IR spectrum, and C) Raman spectrum for GC.

GC structure was analyzed using ^1^H nuclear magnetic resonance spectroscopy (^1^H NMR) and Fourier transform infrared spectroscopy (FT‐IR) (**Figure** **1**A,B). The ^1^H NMR spectrum of GC showed characteristic l‐Cys peaks (3.05–3.45 ppm), whereas disappearance of l‐Cys CHN signal (originally located at 4.02–4.18 ppm) indicated that primary amino groups may have reacted with G4.5 carboxylic groups.^35^ FT‐IR confirmed conjugation of the dendrimer with l‐Cys. G4.5 dendrimers are characterized by C=O stretching vibrations (1650 cm^−1^), NH stretching (3272 cm^−1^), and NH bending (1570 cm^−1^). As activated carboxyl groups reacted with amino groups of l‐Cys, forming amide bonds,^36^ C=O stretching, NH bending, and NH stretching peaks were slightly shifted. Additionally, S—H stretching vibration of l‐Cys (intense absorption at 2550 cm^−1^)^37^ was absent in GC, indicating formation of disulfide bonds, with Raman bands in the fingerprint region (strong sharp band near 500 cm^−1^)^38^ supporting this finding (Figure 1C). These data demonstrate successful conjugation of the G4.5 dendrimer and l‐Cys.

### 2.2 Particle Size and Zeta Potential

**Figure 2:**
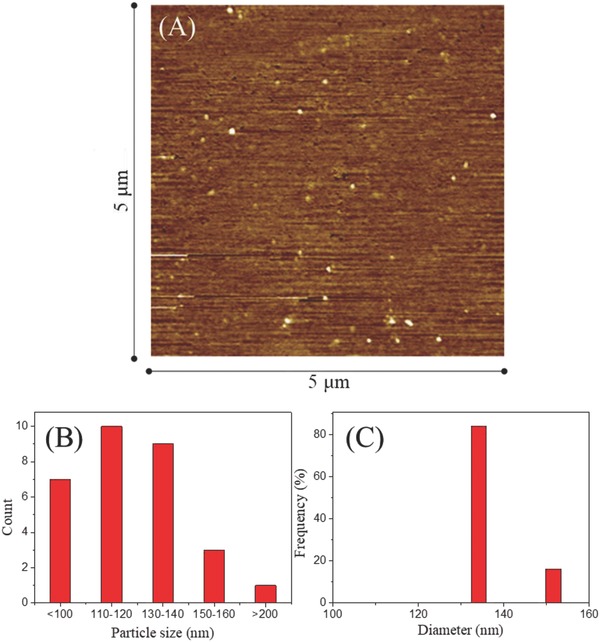
GC particle size. A) AFM‐determined particle size and B) particle size distribution, and C) DLS‐determined particle size.

Dendrimers adopt native (tighter) or denaturated (extended) conformations, depending on functional groups present at the dendrimer surface.^39^ G4.5 dendrimers exhibited pH‐related conformational changes (**Table** **1**). Low ζ‐potential (−56.5) was observed for G4.5 dendrimer at pH 10, with electrostatic repulsion between negative charges forcing the surface groups apart, resulting in an extended conformation of G4.5, with a highly expanded surface area and increased particle size.^40^ After purification (acidification and dialysis), isoelectric point was reached, promoting dense core conformation,^40^ with close to neutral surface potential and a particle size of ≈3.85 nm.^41^ As G4.5 conformation depends on solvent pH, G4.5 can be considered pH‐responsive. GC displayed spherical particles (**Figure** **2**A), 100–200 nm in size (Figure 2B). GC particle size determined with DLS (Z‐average 134.16 nm) was larger than particle size determined using atomic force microscopy (AFM) (average size 125.65 nm). As 100–200‐nm nanoparticles have been shown to extravasate via vascular fenestrations of tumors (the enhanced permeation and retention effect) and avoid uptake by the mononuclear phagocyte system,^42^ GC particle size was considered appropriate for anticancer drug delivery.

**Table 1:** Surface potentials and particle sizes of the G4.5 dendrimer and GC at different pH values

Sample	ζ‐potential [mV]	Particle size [nm]	PI
G4.5(—COO^−^Na^+^)	−56.5 ± 0.2	23.8 ± 2.0	0.38 ± 0.05
G4.5(—COOH)	−0.1	3.85 ± 0.49	0.428
G4.5–Cys	−0.25 ± 0.53	134.16	0.403

### 2.3 Cytotoxicity of the G4.5 and GC Nanocarriers

**Figure 3:**
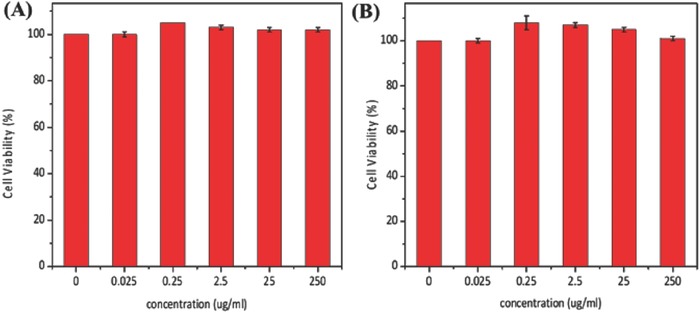
Cytotoxicity at different concentrations from 0.025–250 µg mL^−1^, 24 h exposure A) G4.5, B) GC for Wi‐38 cell, without drug in the section.

Cytotoxicity is a critical consideration for biomaterials. Cytotoxicity of unmodified G4.5 and GC was assessed using the MTT assay in Wi‐38 cell.^43^ Anionic dendrimers were reported to be nontoxic in vitro.^44^, ^45^ In this study, the cell viability of G4.5 and GC is respectably high and biocompatible (24 h exposure) in the concentration range of 0.025–250 µg mL^−1^ and was shown to be nontoxic (<250 µg mL^−1^, 24 h exposure) in Wi‐38 cells, according to the MTT assay (**Figure** **3**A,B). These data indicate that modified GC dendrimers are biocompatible and suitable for external stimuli‐mediated drug delivery.

### 2.4 DOX Loading of Modified Dendrimer Carriers

**Figure 4:**
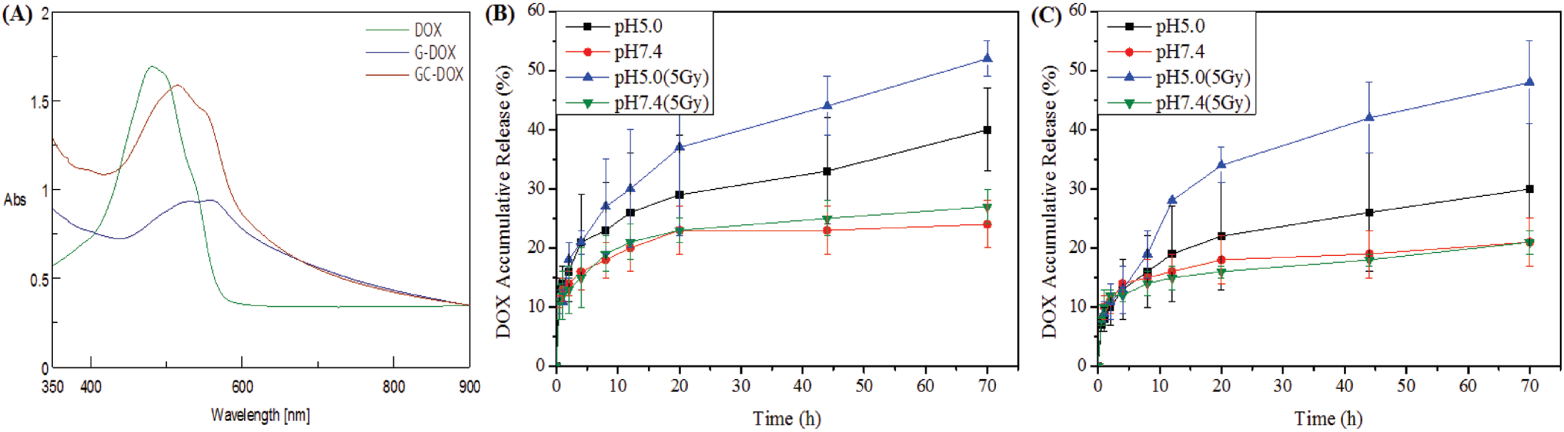
A) UV–vis spectra of free DOX dissolved in phosphate buffered saline (PBS), G4.5/DOX, and GC/DOX. In vitro DOX release profiles from B) G4.5/DOX and C) GC/DOX.

G4.5 and GC DOX payload was analyzed using UV–vis spectroscopy as illustrated in **Figure** **4**A. Typical absorption peak of free DOX at 480 nm^41^, ^46^ showed a red shift after encapsulation.^47^ **Table** **2** summarizes DOX encapsulation efficiency and loading capacity of the analyzed dendrimers, confirming successful loading. Increasing initial DOX concentration improved encapsulation efficiency (up to 38 ± 9.9 and 39 ± 4.2% for G4.5 and GC, respectively). Furthermore, l‐Cys modification of the dendrimer slightly increased DOX loading capacity.

**Table 2:** Encapsulation efficiency (EE) and loading capacity (LC) of dendrimer derivatives

Dendrimer/DOX ratio	LC [%]	EE [%]
G4.5/DOX 1:4.5	2.37	24
GC/DOX 1:4.5	2.44	25
G4.5/DOX 1:9	7.03 ± 1.7	38 ± 9.9
GC/DOX 1:9	7.22 ± 0.7	39 ± 4.2

### 2.5 Drug Release Kinetics

In order to evaluate γ‐radiation (external stimulus) and pH (internal stimulus) response‐mediated drug release from dendrimer derivatives, drug release studies were performed at different pH values (7.4 and 5.0) in the presence and absence of γ‐radiation (5 Gy) for 70 h. DOX release followed a biphasic pattern; initial fast release was followed by long‐term sustained release (Figure 4B,C), in agreement with previous reports.^48^ Drugs were effectively released from pH‐responsive dendrimers, with an increase in G4.5 drug release of ≈16% under acidic conditions (corresponding to endosome and lysosome compartments) compared to drug release at the physiological pH of 7.4.^49^ However, DOX release from the GC carrier only increased by 9%, possibly because the l‐Cys disulfide cross‐linking network on dendrimer surface inhibited release, as this network reduces dendrimer conformational changes.^50^ Moreover, we analyzed free thiol content using Ellman's assay (**Table** **3**). Chromogenic reagent used for thiol detection was 5,5′‐dithiobis‐(2‐nitrobenzoic) acid (DTNB; also known as Ellman's reagent), which is a water‐soluble compound for quantifying free sulfhydryl groups in solution.^51^ l‐Cys was used as a standard in Ellman's assay. Thiol content of GC nanoparticles was also determined in the presence of 5 Gy γ‐radiation. Free thiol concentration was 70 × 10^−6^ m at pH 7.4 in GC phosphate buffered saline (PBS) solution in the absence of radiation, far lower than l‐Cys concentration (8000 × 10^−6^ m), suggesting formation of disulfide bonds in the GC nanoparticle. Furthermore, thiol concentration increased at both pH values (5 and 7.4) after γ‐ray irradiation, indicating partial disulfide bond cleavage in GC particles.

**Table 3:** Concentration of thiol groups in GC at different pH values in the presence of 5 Gy radiation (Ellman's assay, 8000 × 10^−6^ m l‐Cys)

GC thiol content [× 10^−6^ m]		
	0 Gy	5 Gy
pH 5	–	76.6
pH 7.4	70.5	77.1
pH 10	–	68.5

Effects of γ‐radiation on drug release from unmodified G4.5 and GC, were previously studied.^52^ Figure 4B,C illustrates that compared with drug release at acidic pH alone, DOX is more effectively released from G4.5 and GC (increase of 12% and 18%, respectively) when acidic pH is combined with γ‐radiation, possibly because of GC disulfide bond cleavage by radiation and conformational changes of the dendrimer in acidic environment.^53^, ^54^, ^55^ Taken together, these results indicate that GC nanocarriers combined with γ‐radiation provide a platform for release of therapeutic agents under acidic conditions of the tumor microenvironment.

### 2.6 Morphological Changes in HeLa Cells Caused by Gamma Radiation

**Figure 5:**
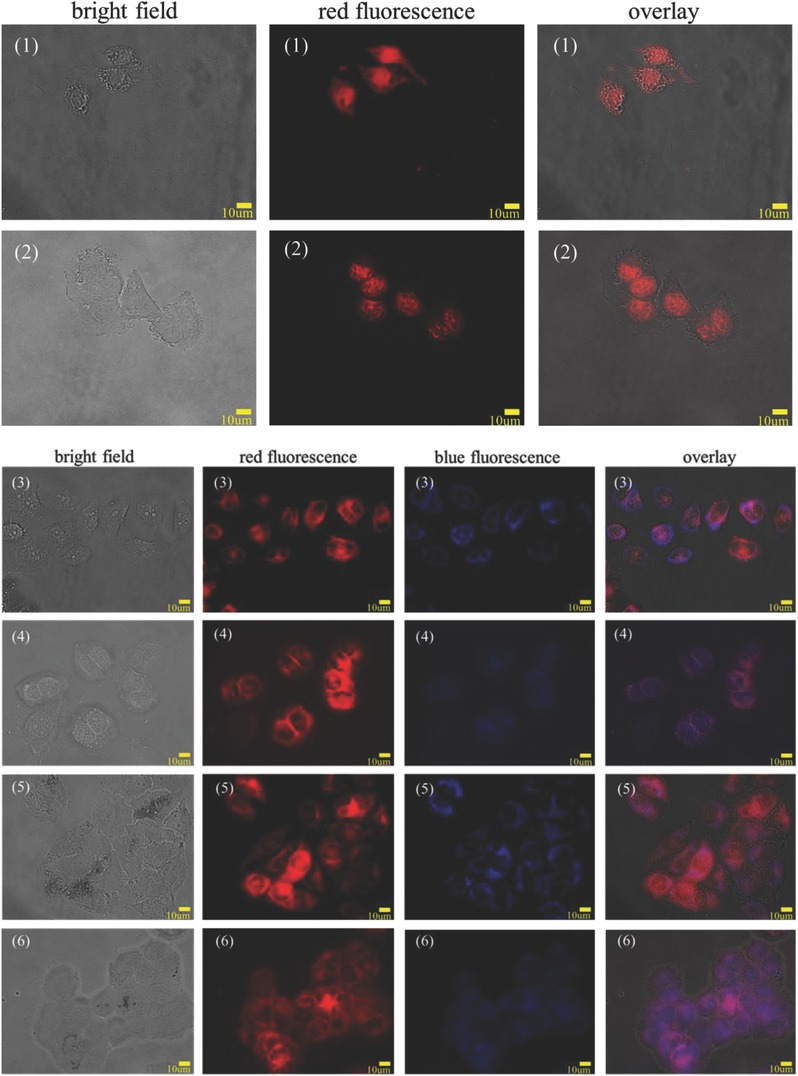
Fluorescence microscopy images of HeLa cells treated with free DOX, G4.5/DOX, and GC/DOX for 8 h; (1) free DOX, (2) free DOX + 5 Gy, (3) G4.5/DOX, (4) G4.5/DOX + 5 Gy, (5) GC/DOX, (6) GC/DOX + 5 Gy. G4.5 dendrimers (blue); DOX (red).

Radiation energy is deposited in cell nuclei, elicits mutagenic or clastogenic effects, and causes damage.^56^ Radiation energy is deposited in cell nuclei, elicits mutagenic or clastogenic effects, and causes damage.^57^ We confirmed dendrimer carrier uptake by HeLa cells and observed significant drug presence in the nucleus (**Figure** **5**). After irradiation, micronucleus formation^56^ and cell lysis^58^ were observed. Additionally, cancer cells were reported to exhibit functional deficiency and reduced proliferation potential in response to γ‐radiation.^59^, ^60^

**Figure 6:**
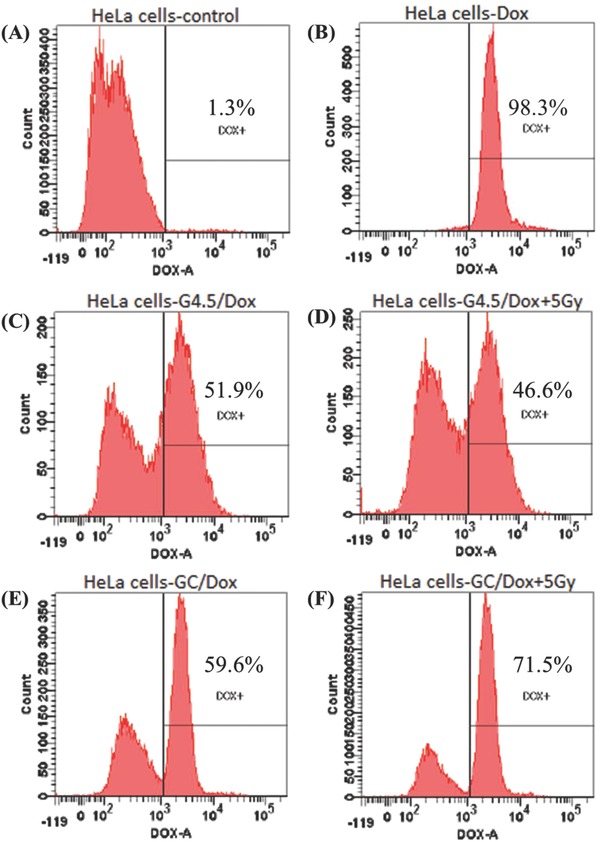
Flow cytometry analysis of HeLa cells treated with A) PBS (control), B) free DOX, C) G4.5/DOX, D) G4.5/DOX + 5 Gy, E) GC/DOX, and F) GC/DOX + 5 Gy for 12 h.

Cellular uptake of free DOX and DOX‐loaded G4.5 derivatives in the presence and absence of radiation was quantified using flow cytometry. Control exhibited no fluorescence, whereas free DOX, despite diffusion across the cell membrane, showed relatively high cytotoxicity (**Figure** **6**B). Cells treated with G4.5/DOX and GC/DOX exhibited strong fluorescence. Cellular uptake of GC/DOX was significantly higher than that of G4.5/DOX, possibly because of high affinity of l‐Cys for cells.^61^

### 2.7 In Vitro Cytotoxicity

**Figure 7:**
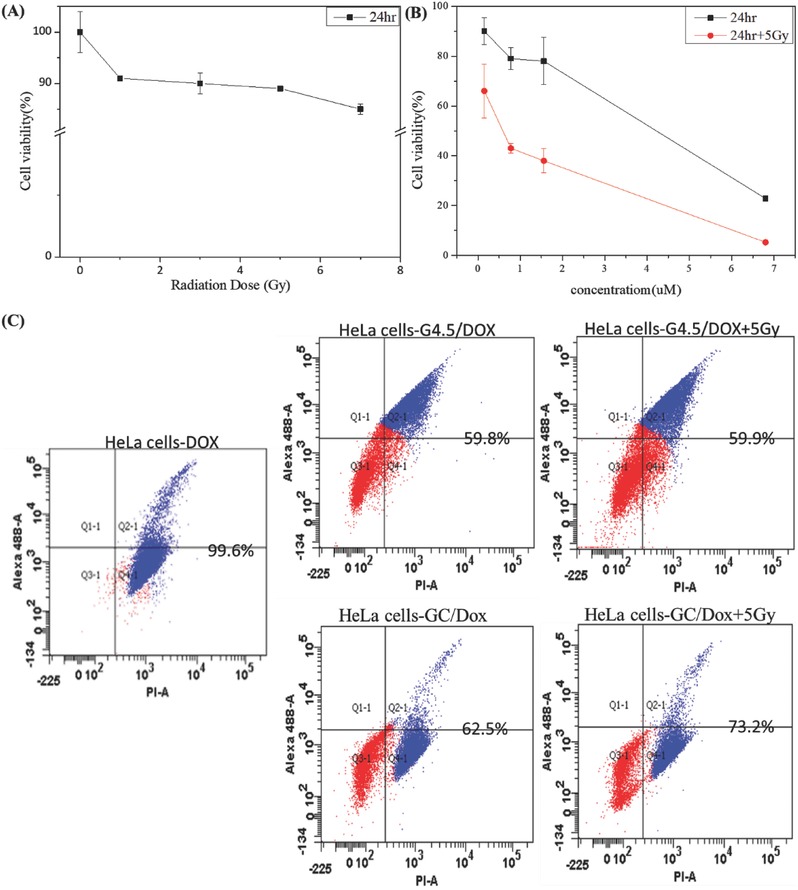
In vitro cytotoxicity. A) Exposure of HeLa cells to γ‐radiation. B) Effects of free DOX and combined DOX/γ‐radiation treatment on proliferation of HeLa cells (*n* = 3). C) Apoptosis and necrosis determined using flow cytometry and annexin V/propidium iodide staining; HeLa cells were treated with free DOX, G4.5/DOX, and GC/DOX for 12 h, followed by exposure to γ‐radiation (5 Gy).

Exposure to γ‐radiation decreased proliferation of HeLa cells in a dose‐dependent manner, as previously reported.^62^, ^63^, ^64^ Cell viability was 91%, 90%, 89%, and 85% after exposure to 1, 3, 5, and 7 Gy, respectively. DOX is an effective chemotherapeutic drug and reduced cell viability, as expected. Survival rates of HeLa cells after treatment with free DOX alone or in combination with 5 Gy of γ‐radiation are shown in **Figure** **7**B,C. Treatment with 0.015 × 10^−6^, 0.15 × 10^−6^, 0.78 × 10^−6^, and 1.56 × 10^−6^ m of DOX caused directly proportional reduction in cell proliferation after 24 h. Compared to lower treatment concentrations of free DOX, 11 × 10^−6^ m DOX treatment resulted in extremely high inhibition of HeLa cells proliferation after 12 h. Combined chemotherapy and radiotherapy treatment enhanced inhibition of cell proliferation,^65^ with cell viability of 71%, 66%, 43%, 38%, and 5.2% after incubation with various concentrations of DOX and exposure to γ‐radiation. Synergistic effects of DOX and γ‐radiation led to significantly higher cytotoxicity, compared with DOX treatment alone. These results suggest that therapeutic strategies combining low concentrations of anticancer drugs and low doses of γ‐radiation can markedly decrease cancer cell proliferation.

Therapeutic effects of anticancer drugs are accompanied by damage to healthy cells. By providing effective targeted drug delivery, DOX‐loaded dendrimers and GC nanoparticles display lower cytotoxicity compared to free DOX treatment. These nanocarriers were confirmed to be non‐toxic at a concentration of 0.25 mg mL^−1^ using the MTT assay. Additionally, cell death was assessed using flow cytometry and annexin V–propidium iodide (PI) staining. Cell viability after GC/DOX treatment was lower than viability after G4.5/DOX treatment, in the presence and absence of γ‐radiation (Figure 7C), as high cellular affinity of l‐Cys (in GC/DOX) led to a higher uptake of GC/DOX.^61^ Additionally, in the presence of γ‐radiation, disulfide bonds of GC/DOX were effectively lysed by radiation‐induced free radicals, resulting in enhanced drug release.^54^ These in vitro data suggest that GC/DOX is a promising carrier for external stimuli‐mediated drug release. To further confirm that radiation exposure enhances drug release from GC/DOX, an in vivo zebrafish study was performed.

### 2.8 In Vivo Zebrafish Experiments

**Figure 8:**
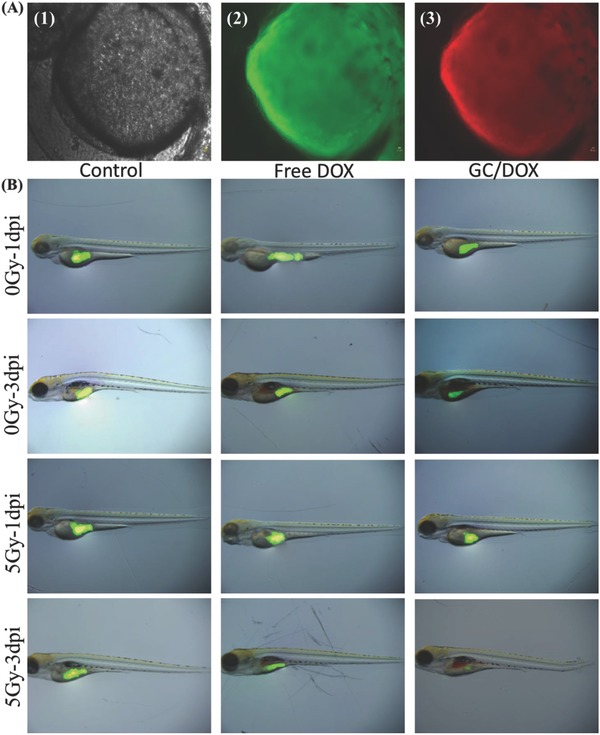
In vivo zebrafish study. A) 2‐dpf zebrafish were exposed to GC/DOX for 24 h; bright‐field microscopy (1), green fluorescence (2), and red fluorescence (3). B) HeLa cells were labeled with CFSE and treated with free DOX and GC/DOX for 48 h. CFSE fluorescence was recorded 0 and 3 dpi (in the presence and absence of γ‐radiation).

**Figure 9:**
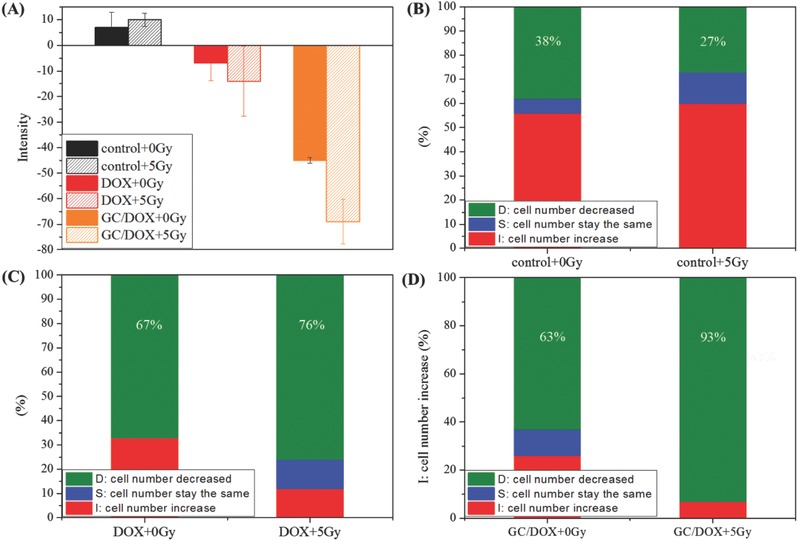
CFSE fluorescence. A) Average CFSE fluorescence. The zebrafish were treated with PBS, free DOX, and GC/DOX with and without 5 Gy (20 zebrafish per group). B) Survival rate of HeLa cells in zebrafish treated with B) PBS C) free DOX D) GC/DOX in the absence or presence of 5 Gy radiation for 48 h (20 zebrafish per group).

In vivo effects of external and internal stimuli on GC drug delivery were analyzed using transparent zebrafish embryos and noninvasive imaging of fluorescent G4.5 and DOX.^66^ After 24 h exposure of 2 dpf zebrafish embryos to GC/DOX, fluorescence detected in the eyes was rather weak, with green G4.5 and red DOX fluorescence mostly accumulating in the embryo bodies and tails of the zebrafish (**Figure** **8**A). Survival and morphologic appearance of the embryos exposed to γ‐radiation (5 Gy) were monitored using carboxyfluorescein succinimidyl ester (CFSE) labeling. CFSE is used for cell tracking and proliferation studies, including HeLa cell viability assays.^67^, ^68^ Control group showed clear weak‐intensity fluorescence. **Figure** **9**A shows positive average CFSE fluorescence intensity in the control group, indicating increased number of cancer cells. Decrease in the intensity of fluorescence (negative values compared to control) corresponds to cell death and suggests drug‐induced cytotoxicity. Accordingly, when combined with radiation, GC successfully delivered DOX to cancer cells and inhibited cell proliferation. In 2 dpf embryos, chemotherapy combined with 5 Gy γ‐irradiation profoundly affected the number of HeLa cells: CFSE fluorescence of >5%, 0–5%, and decreased fluorescence compared to control led to increased, unchanged, and decreased number of HeLa cells, respectively (Figure 9B–D). In G4.5/DOX and GC/DOX groups, number of HeLa cells decreased, compared to control. When free DOX and GC/DOX groups were exposed to 5 Gy γ‐radiation, HeLa cells proliferation was substantially more suppressed. Furthermore, proportion of surviving HeLa cells after radiation treatment (5 Gy) in GC/DOX group was lower than in free DOX group, a finding supporting synergistic effects of GC‐delivered chemotherapy and radiation.

## 3 Conclusions

This study evaluated drug release from G4.5 and GC nanocarriers using pH and γ‐radiation exposure as external stimuli. G4.5 and GC carriers effectively encapsulated anticancer drug DOX. Radiation‐generated ROS induced conformational changes in the dendrimer internal structure and cleaved the GC disulfide network, resulting in radiation‐triggered higher drug release from GC than from G4.5 nanocarriers under acidic conditions. Moreover, G4.5/DOX and GC/DOX were successfully internalized by HeLa cells, delivering DOX to the nucleus. GC/DOX treatment combined with radiation successfully inhibited HeLa cells proliferation in vitro and in vivo in zebrafish. Radiation‐mediated release of DOX from GC and enhanced GC cellular uptake support this carrier as a promising drug delivery system for local chemotherapy.

## 4 Experimental Section

**Scheme 2:**
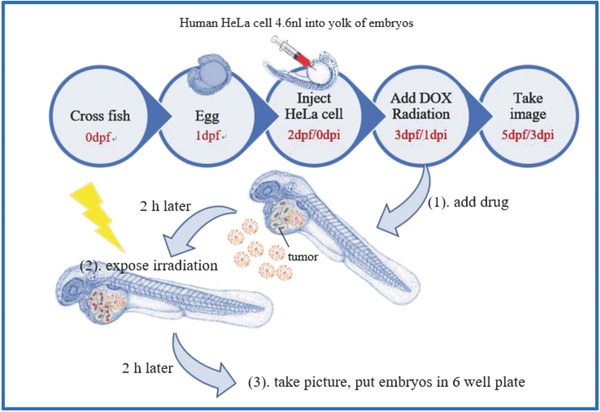
Embryos were replaced in G 4.5/DOX ad GC/DOX with absence or presence of radiation.

*Synthesis of G4.5‐Cys Nanoparticles*: The G4.5 PAMAM dendrimer was modified with l‐Cys to form a functional GC nanocarrier through amide bonds, as depicted in **Scheme** **2**. Briefly, to prepare the G4.5 solutions, the sodium in G4.5–COO^−^Na^+^ was removed through acidification with 1 n hydrochloric acid up to a pH of 5, followed by dialysis with water. Excess NHS and EDC were added to activate the carboxylic acid groups of G4.5 dendrimers overnight. The activated dendrimer solution was then reacted slowly with l‐Cys (mole ratio of 1:320) for 24 h. Finally, the reagent solutions were dialyzed (molecular weight cutoff [MWCO]:6–8 kDa) with ultradistilled water to remove the unbound molecules. Lastly, the solution was lyophilized overnight for the characterization of the GC nanocarrier, which was confirmed through FT‐IR (PerkinElmer) and NMR (Bruker Avance III HD600‐MHz NMR with D_2_O as the solvent).^30^, ^69^ The morphology and the particle size were confirmed by atomic force microscopy (Park NX10)

*Dynamic Light Scattering and ζ‐Potential*: The hydrodynamic size and zeta potential of the dendrimer derivatives were analyzed using the SZ‐100 nanoparticle analyzer (Horiba). Functional G4.5 dendrimers and GC nanocarriers were prepared in ultradistilled water and assessed using a 0.45 µm filter. Following three measurements, the average hydrodynamic diameter and zeta potential were determined at 25 °C and the scattering angle at 90°.

*Free Thiol Determination by Ellman's Assay*: The DNTB solution was preparing with 50 × 10^−3^ m sodium acetate and 2 × 10^−3^ m DTNB in ultradistilled water and adjusted solution to corresponding pH (5.0, 7.4, and 10.0). Mix the DTNB reagent by adding 50 µL of the DTNB solution, 100 µL of Tris solution, and 840 µL of ultradistilled water. (Final volume will be 1000 µL with a 10 µL each sample.) GC nanoparticle (powder) was added to aforementioned solution based on the concentration of l‐cysteine fixed at 8000 × 10^−6^ m. Finally, the optical absorbance was measured at 412 nm, with a standard SH (l‐cysteine) as a calibration curve.

*In Vitro Cytotoxicity*: Human cervical carcinoma (HeLa) cells were grown in Dulbecco's modified Eagle's medium and supplemented with 10% fetal bovine serum and 1% sodium pyruvate at 37 °C and 5% CO_2_. To evaluate the cytotoxicity of the G4.5 and GC carriers through flow cytometry (Becton Dickinson FACS Area III cell sorter), the cells were seeded onto T25 plates at a density of 1 × 10^6^ cells per well and were grown overnight. Subsequently, the medium was substituted with the control (medium), G4.5, and GC at 0.25mg mL^−1^.^45^ After 12 h, the cells were washed, trypsinized, centrifuged, and collected. In addition, Alexa Fluor488 annexin V and PI were added, according to the manufacturer's instructions. Briefly, the cells were suspended in 500 µL of 1× annexin‐binding buffer for analysis.

*Loading of DOX Onto G4.5 and G4.5‐Cys*: DOX hydrochloride (DOX·HCl) was dissolved in dimethyl sulfoxide (DMSO) and neutralized with excess diisopropyl ethylamine to generate hydrophobic molecules.^48^ Followed by slow dropping into the dendrimer complex solution, which was vigorously stirred overnight. The resulting solution was placed under dialysis in PBS for 24 h to remove the excess DOX.^41^, ^46^, ^70^ Dendrimer–drug interactions were evaluated through UV–vis spectrophotometer (Jasco V‐730 UV–vis spectrophotometer). The calibration curve of free DOX was acquired at 500 nm. Furthermore, a linear relationship was observed for the concentration of DOX in the range of 0.03 × 10^−3^ to 1.72 × 10^−3^ m, with a correlation coefficient of 0.9986. The loading capacity (LC) and encapsulation efficiency (EE) were obtained by the following equations^70^

(1) $$\mathrm{Encapsulation}\;\mathrm{efficiency}\;\left(\mathrm{EE}\right)\;\left(\%\right)\;=\;\frac{\mathrm{Amount}\;\mathrm{of}\;\mathrm{drug}\;\mathrm{in}\;\mathrm{carrier}}{\mathrm{Initial}\;\mathrm{amount}\;\mathrm{of}\;\mathrm{drug}\;\mathrm{used}\;\mathrm{for}\;\mathrm{loading}}\;\times \;100\%$$

(2) $$\mathrm{Loading}\;\mathrm{capacity}\;\left(\mathrm{LC}\right)\;\left(\%\right)\;=\;\frac{\mathrm{drug}\;\mathrm{weight}\;\mathrm{in}\;\mathrm{the}\;\mathrm{carrier}}{\mathrm{weight}\;\mathrm{of}\;\mathrm{carrier}}\;\times \;100\%$$

*In Vitro Drug Release Kinetics*: A dialysis method was used to assess the release of DOX from functional G4.5 derivatives at different pH values and in the absence or presence of gamma radiation. Briefly, G4.5/DOX and GC/DOX solutions (for each solution type, one group was exposed to 5 Gy gamma radiation before release) were placed in dialysis membranes (MWCO 6–8 KDa) with a phosphate buffer (pH7.4) and citrate buffer(pH5.0) under reservoir‐sink conditions at 37 °C, with constant stirring. The experiment was performed in triplicate. At each time interval, 2 mL of the buffer was collected, and the absorbance was measured at 500 nm. Furthermore, the volume of the outer phase buffer was kept constant by replenishing with the corresponding fresh buffer solution used in the system, according to previous protocols.^65^, ^71^

(3) $$\mathrm{Cumulative}\;\mathrm{release}\;\left(\%\right)\;=\;\frac{\mathrm{Concentration}\;\mathrm{of}\;\mathrm{drug}\;\mathrm{release}}{\mathrm{Concentration}\;\mathrm{of}\;\mathrm{drug}\;\mathrm{load}}\;\times \;100\%$$

*In Vitro Cellular Uptake Studies*: The cells were incubated in media with free DOX, G4.5/DOX, and GC/DOX for 8 h, with or without exposure to 5 Gy gamma radiation. After gamma‐ray exposure (ELEKTA Synergy), the cells were continuously cultured for 5 h and then washed and viewed through fluorescence microscopy (iRiSTMDigital Cell Imaging System).

*Drug and Radiation Treatment*: HeLa cells (6 × 10^4^) were seeded in a 35 × 10 mm dish. The next day, free DOX (at 6.8 × 10^−6^, 1.56 × 10^−6^, 0.78 × 10^−6^, 0.15 × 10^−6^, and 0.015 × 10^−6^ m) alone or in combination with radiation was added to the cells for 24 h. The cells were treated or untreated with various doses of radiation (1, 3, 5, and 7 Gy) and subsequently cultured for 24 h. After incubation, the cell viability was assessed through an MTT assay. The 50 µL of DMSO was placed to dissolve the formazan crystals after medium was replaced with an MTT solution (0.5 mg mL^−1^) for 4 h. Finally, the absorbance (570 nm) was measured through ELISA (ELISA Reader, Thermo Multiskan FC Microplate Photometer), according to a previous protocol.^62^ The cell viability was calculated as follows

(4) $$\mathrm{Cell}\;\mathrm{viability}\;\left(\%\right)\;=\;\frac{\mathrm{absorbance}\;\mathrm{of}\;\mathrm{treated}\;\mathrm{cells}}{\mathrm{absorbance}\;\mathrm{of}\;\mathrm{control}\;\mathrm{cells}}\;\times \;100\%$$

*Flow Cytometry*: HeLa cells were treated with 11 × 10^−6^ m of G4.5/DOX and GC/DOX for 12 h in the presence or absence of gamma radiation. Alexa Fluor488 annexin V and PI were added to the cells according to the manufacturer's instructions. In addition, the cells were washed, centrifuged, and suspended in 500 µL of 1× annexin‐binding buffer for analysis.

*In Vivo Study in Zebrafish*: Zebrafish from the National Health Research Institutes were used to evaluate the effects of chemotherapy combined with radiotherapy in an in vivo system. HeLa cell lines (200 cells) were labeled with CFSE and injected into the yolk space of 2‐d‐old zebrafish embryos (i.e.,embryos at 0–3 d post fertilization [dpf]), according to a previous protocol.^72^ Eight groups of 20 zebrafish each were examined. The three dpfblastulas were immersed in a solution containing 2 × 10^−6^ m of free DOX, G4.5/DOX, and GC/DOX. After 2 h, the embryos were replaced with PBS in six‐well plates in the absence or presence of radiation, and images were captured at 1 dpi. Subsequently, the embryos were immersed in DOX‐loaded dendrimer derivatives and incubated for 48 h. Finally, images were captured again at 3 dpi^73^ and the intensity was calculated as follows

(5) $$\mathrm{Intensity}\;\left(\%\right)=\;\frac{\mathrm{Fluorescence}\;\mathrm{area}\;\mathrm{of}\;\left(3\;\mathrm{dpi}-1\;\mathrm{dpi}\right)}{\mathrm{Fluorescence}\;\mathrm{area}\;\mathrm{of}\;1\;\mathrm{dpi}}\;\times \;100\%$$

## Conflict of Interest

The authors declare no conflict of interest.
